# Claudius Galenus and His System

**Published:** 1908-03-07

**Authors:** 


### The Evolution of Medicine.

### CLAUDIUS GALENUS AND HIS SYSTEM.

The Galenic system of medicine is a compromise between pure scientific medicine and philosophic, empiric Hippocratism with its contradictions and anomalies. In many ways profoundly interesting, it gives us a glimpse into the character of its founder. Galen was an Aristotelian, not indeed to the core, for he had a sneaking regard for Plato, but sufficiently to make the philosophy of the son of Nicomachus the guiding principle in his theories. To the thoughtful reader who opens for the first time the "De Locis Affectis" the revelation which it gives of the mental attitude of its author is indeed surprising.

The faults of the system founded by Galen are many. It is a combination, so far as such is possible, of the terse concrete empiricism of the physician of Cos with the vague abstractions of the pupils of Socrates. It is less a system than a creed. It was founded on empiricism and buttressed by speciosity of argument and explanation. It was a huge fault which the experience and sagacity of years could not entirely remedy. As a philosopher, Galen was impossible; as a theoretical physician he was absurd. But, for all that, reading his works in the light of modern knowledge, who can fail to be struck by the magnitude of the task which he accomplished? A work from which ordinary men would have shrunk he carried out in the leisure hours of an onerous and exacting practice.

He taught that there exists no sharp line of demarcation between health and disease. Both are actual results of the relation of the human body to external influences, acting upon the organism. To these influences he adds the influence of the various temperaments, generated in the normal condition from the preponderance of elementary substances, and divided into the plethoric, choleric, melancholic, and phlegmatic varieties. So long as external influences react "naturally," a condition of health results; when the reaction is unnatural, disease supervenes. Symptomatology he divides into three main groups (*a*) special disturbance of function, (*b*) consecutive appearances (τὰ συμβεβηκότα τοῖς σώμασι), and finally (*c*) changes in secretions and excretions. Primary disturbance alone is pathognomonic; the others are adventitious. Disturbance, in fine, is something in process of generation; disease is something already generated, mainly manifesting itself in disturbance of function and appreciable change in the structure of parts.

He accordingly divides his diseases into affections of primary substances of tissues (which are again subdivided into (1) departures from the normal mechanical state, and (2) changes in the essential qualities of the tissues) and of regional pathology, which latter he classes as alterations of structure, number, surroundings, size, and relations.

In his treatment Galen was conservative. He agreed with Hippocrates that "the primary condition is not to damage." "I know," he writes in his Commentary on the works of the master, "that there is a deal of truth underlying the dictum 'Be useful but do no injury.' When it happens that by the use of some drastic remedy we lose our patient, we shall speedily understand its significance." In some chronic cases he is prepared to allow the physician to attempt a cure "by contraries." On other occasions the homœopathic rule is applied with the proviso, overlooked by Hahnemann, that the remedies which act beneficially in such cases do so not because they cause effects corresponding to the disease, but through "other qualities inherent to them." He employed venesection largely, but wrote three treatises warning against its indiscriminate use. The most important new drug he used was opium. His consumptive patients he sent to Egypt, ordering them a diet of goat's or of human milk, and confining his active treatment to allaying irritation or promoting expectoration by infusions of myrrh, squills, or honey. In cases of phrenitis (typhus) he commenced his treatment by a venesection, following it by cold compresses to the patient's head and the exhibition of narcotics during the stage of delirium. Surgically he added little to the known knowledge of the time. He wrote against puncturing an empyema, or trephining a portion of rib to evacuate the pus—operations which Hippocrates had advised. Both pneumonia and pleurisy he described, and drew attention to the rise of surface temperature on the affected side. Gastric and intestinal diseases he treated by dieting his patients. His works on the kidneys and on the urine rank among his best books.

In the history of medicine Galen deserves a high place. Vaunted on the one hand as an individual of almost divine attainments, flattered by his pupils as the equal of Hippocrates, he lived to hear his courage called into question by his contemporaries, his theories impugned by his colleagues, and his vanity and conceit made the butt of laymen. When contemporary opinion on his merits is so dissonant, who to-day shall gauge the depth of his littleness or calculate the height of his greatness? The measures by which we can estimate him are his extant works. With all its faults, his system is a gigantic, a monumental, work which no ordinary man could have achieved. We may minimise its importance, we may call it stereotyped, artificial, empirical, vague, and we may regret that its influence on medical science has been reactionary and retrogressive, calculated to retard advance rather than to promote it, but at any rate we must acknowledge the skill with which it was drawn up and the profundity of learning with which it was supported. As a physician and teacher, Galen's merits outweigh his shortcomings. He saw clearly, but his range of vision was limited. He gave to medicine a system which was destined to influence centuries and generations, only to collapse ignominously when the very facts that he had before him when he devised it became better known and more worthily interpreted.

